# A novel *APC* mosaicism in a patient with familial adenomatous polyposis

**DOI:** 10.1038/hgv.2015.57

**Published:** 2015-12-10

**Authors:** Moriya Iwaizumi, Hong Tao, Kiyoshi Yamaguchi, Hidetaka Yamada, Kazuya Shinmura, Tomoaki Kahyo, Yoshiyuki Yamanaka, Kiyotaka Kurachi, Ken Sugimoto, Yoichi Furukawa, Haruhiko Sugimura

**Affiliations:** 1 First Department of Medicine, Hamamatsu University School of Medicine, Shizuoka, Japan; 2 Department of Tumor Pathology, Hamamatsu University School of Medicine, Shizuoka, Japan; 3 Division of Clinical Genome Research, Advanced Clinical Research Center, Institute of Medical Science, The University of Tokyo, Tokyo, Japan; 4 Second Department of Medicine, Hamamatsu University School of Medicine, Shizuoka, Japan

## Abstract

Using next-generation sequencing (NGS) to analyze a patient with sporadic familial adenomatous polyposis in whom no *APC* mutations were found by Sanger sequencing, we identified a novel *APC* mosaicism at a spliced donor site (c.834+2 T>C) in his leukocytes, normal colonic mucosa and adenoma. The detection of *APC* mosaicism using NGS can be useful in providing appropriate genetic counseling and surveillance of at risk family members as well as the proband.

Familial adenomatous polyposis (FAP) is an autosomal dominant colorectal tumor syndrome characterized by the formation of a number of adenomatous polyps throughout the entire colon. Clinically, a precise family history and genetic counseling together with surveillance of the colon and extra-colonic organs for people at risk is important to prevent cancer progression, because of the almost 100% lifetime risk of colorectal cancer if the colon is not removed. Although genetic testing for a germline mutation in the *APC* tumor-suppressor gene is typically performed using conventional Sanger sequencing, sometimes pathogenic *APC* mutations cannot be detected because (i) the testing region is not routinely included in the 3′-half, promoter region, or 5′- or 3′- UTR, (ii) it is hard to identify large deletions/insertions using the Sanger method, (iii) some cases of adenomatous polyposis are caused by *MUTYH*, *POLD1* or *POLE* mutations,^1,2^ and (iv) some FAP cases arise from somatic *APC* mosaicism.^3^ Technology for DNA sequencing has recently made rapid progress. Based on the deep-sequencing method, various types of next-generation sequencing (NGS) have provided a vast amount of genomic information with high sensitivity and at a low cost, such that NGS is now an indispensable tool.^4^ Deep sequencing can be categorized as follows: (1) whole-genome sequencing, (2) whole-exome sequencing, (3) target sequencing for the validation of a mutation identified by whole-exome sequencing and (4) transcriptome sequencing.^5^ Although it is convenient to use whole-genome sequencing when searching for an unknown variant, target resequencing may be of great use when a patient has a typical clinical phenotype of a hereditary disease with a known causative gene mutation but has neither a family history nor any pathogenic alteration detected using Sanger sequencing or the multiple ligation-dependent probe amplification (MLPA) method. We explain here how we detected a novel somatic mosaicism in the *APC* gene in a patient with sporadic FAP using NGS.

This project was approved by the Institutional Review Board of Hamamatsu University School of Medicine (23–91), and written informed consent was obtained from the patient and his parents. For the initial mutation analysis by Sanger sequencing, peripheral blood samples were obtained from the proband and DNA was extracted. All the coding exons of *MUTYH* and *APC* and their boundary regions were amplified using PCR and directly sequenced using the ABI BigDye Terminator Ready Reaction Mix (Applied Biosystems, Foster City, CA) and an ABI3100 Genetic Analyzer (Applied Biosystems).^6^ For the MLPA analysis, an MLPA kit (P043 APC) was purchased from MRC-Holland (Amsterdam, The Netherlands), and the reactions were carried out according to the manufacturer’s instructions.^7^ For targeted resequencing, all coding exons of *APC* and their boundary regions were amplified using PCR. Amplicon sequencing was carried out using the IonPGM Sequencing 400 kit (Thermo Fischer Scientific, Waltham, MA). To confirm mosaicism, genomic DNA was extracted from peripheral blood, non-tumorous colonic mucosa and colonic adenomatous polyps from the proband (AGFAP001-1), as well as from peripheral blood from his parents (his father, AGFAP001-2; his mother, AGFAP001-3). Deep-target resequencing was achieved using the IonPGM sequencing 200 kit (Thermo Fischer Scientific) with libraries of PCR products prepared using the Ion Plus Fragment Library Kit (Thermo Fischer Scientific).^3^ Variants were identified using the variant cellular deployed with Torrent Suite (Thermo Fischer Scientific). For APC messenger RNA transcription analysis, RNA was extracted from proband blood samples with a PAX-gene Blood RNA Kit (QIAGEN, Hilden, Germany) and converted to first-strand complement DNAs using the superscript first-strand synthesis system for reverse transcription (RT)–PCR (Life Technologies, Carlsbad, CA, USA). RT–PCR was performed using the primer set 5′-AGGTCATTGCTTCTGCTG-3′ and 5′-CCAGAAGTTGCCATGTTGAT-3′.

The index case was a 40-year-old man (AGFAP001-1; proband) who visited our hospital because of the results of a fecal occult blood examination for colorectal cancer. Several colorectal adenomatous polyps and fundic gland polyposes in the stomach were identified during an endoscopy, and a total colectomy was performed (Figure 1a). As shown in the pedigree of his family (Figure 1b), he had no family history of colonic polyposis or colorectal cancer despite his typical features of FAP. Because the proband had multiple polyposis typical of FAP but had no family history of polyposis in the gut axis, we next performed a mutational analysis for *MUTYH* and *APC* using Sanger sequencing and a MLPA analysis. We undertook the MLPA analysis because *MUTYH*-associated polyposis (MAP) is an autosomal recessive disease and the proband’s parents may have been carriers of the *MUTYH* mutation. However, we could not detect any pathogenic variants or large insertions/deletions. At this time, we assumed that the proband had some form of germline *APC* mosaicism that could not be detected using conventional genetic testing. To explore the possibility of somatic *APC* mosaicism, we next designed primer sets that covered all the coding exons and their boundary regions and conducted amplicon sequencing. We identified eight polymorphic variants in the coding region and one novel variant in the splicing donor site (c.834+2T>C) of the proband (Table 1). In the screening amplicon sequencing, the number of reference reads (T) in c.834+2T>C was 279 and the number of variant reads (C) was 25 (mutation frequency, 8.1%). To confirm the frequency of the novel variant, we further used deep sequencing of proband DNA isolated from peripheral blood, normal colonic mucosa, and a colonic adenoma. As shown in Table 1, the c.834+2T>C mutation was observed in 26 183 of 206 777 reads (12.7%) in his peripheral blood, whereas his parents did not have mutation reads. Interestingly, we found different frequencies for the c.834+2T>C mutation in the normal colonic mucosa and colonic adenoma of the proband (521 of 3269 reads (15.9%) and 4328 of 18 226 (23.7%) in normal colonic mucosa, and 2399 of 6480 reads (37.0%) and 2184 of 4553 (48.0%) in colonic adenoma, Figure 1c). These data suggest that the c.834+2T>C mutation has a crucial role in the development of adenoma. In a transcription analysis, the c.834+2T>C mutation induced aberrant transcription, as predicted, producing a truncated *APC* protein (Figures 1d and e).

The standard clinical diagnosis of FAP is based on the identification of >100 colorectal adenomatous polyps.^8^ However, when a proband has no family history of an FAP phenotype, we should consider the following possibilities: (1) a *de novo APC* mutation, which is identified in 10–25% of FAP patients; (2) MAP, which is an autosomal recessive hereditary disease; or (3) somatic *APC* mosaicism. Of 242 patients with pathogenic *APC* mutations, Hes *et al.* reported that 10 cases (4%) had somatic *APC* mosaicism based on the results of denaturing gradient gel electrophoresis or a protein truncation test and/or direct sequencing,^9^ but the sensitivities of these methods may be relatively low. As NGS sheds light on the effective detection of somatic mosaicism,^10,11^ we used this technique to identify a novel *APC* somatic mosaicism of the spliced donor site using amplicon sequencing followed by target resequencing. During embryogenesis, the zygote forms three germ layers: the ectoderm, the endoderm, and the mesoderm. Because peripheral blood originates from the mesoderm and the colonic mucosa originates from the endoderm, the mutational event in the proband must have occurred at least before the separation of these two layers, at the latest. Therefore, we speculated that the mutation in our case occurred at an early stage of segmentation, since all the germ layers form at almost the same early stage (~3 weeks) of embryonic development.

How can we make the best use of somatic mosaicism results such as these in clinical practice, especially for patient care? It can be a challenge to convey this complicated information to the proband and his or her descendants. When an ordinary *de novo* germline *APC* mutation is identified in a proband, his or her children have a 50% risk of inheriting a germline *APC* mutation. However, when a proband has <50% somatic mosaicism, as in this case and others, the inheritance risk for any offspring depends on the level of mosaicism in the parental germ cell.^9,12,13^ In this study, although we did not test the *APC* mutation rate of the germ cells (sperm), the recurrence risk for the proband’s children may be low (<50%), judging from the low *APC* mutation prevalence in his normal colonic mucosa, colonic polyps, and peripheral blood. This information may be useful for the decision-making process of the client if he hopes to have more genetic counseling when he wants a child.

In conclusion, we identified a novel somatic *APC* mosaicism in a patient with a mutable allele fraction of 12.7% in his peripheral blood using NGS. Because an understanding of the accurate diagnosis of somatic mosaicism for probands with FAP will be of great use for clinical practice, such as surveillance and genetic counseling, our finding should accelerate the application of NGS in clinical practice.

## Figures and Tables

**Figure 1:**
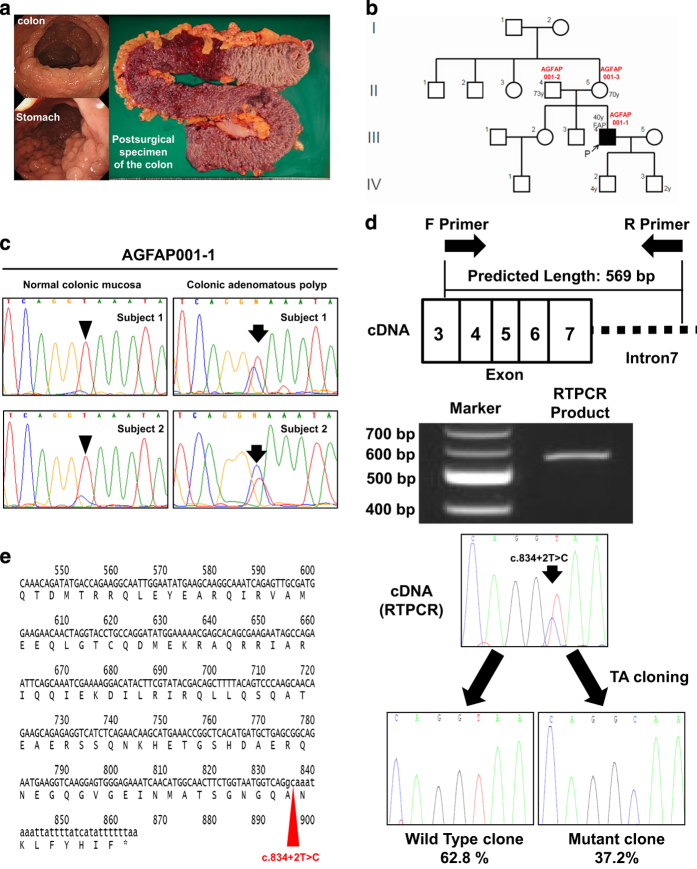
Clinical and genomic features of a case with a novel *APC* mosaicism. (**a**) Colonic adenomatous polyposis and fundic grand polyps of the colon. (**b**) Pedigree chart. DNA from the peripheral lymphocytes of the parent-proband trio (proband, AGFAP001-1; his father, AGFAP001-2; and his mother, AGFAP001-3) was used for the *APC* mutation analysis using deep sequencing. (**c**) Confirmation of the c.834+2T>C mutation by Sanger sequencing using DNA from normal colonic mucosa (subject 1 and 2; arrowheads) and colonic adenomatous polyp (subject 1 and 2; arrows). (**d**) Transcriptional analysis. Because we detected a mosaic mutation at the spliced donor site (Table 1), the mutation was potentially capable of resulting in an extended amino acid translation at intron 7. We detected an RT–PCR product using electrophoresis that was transcribed into an aberrant mRNA with intron 7. (**e**) Direct sequencing followed by TA cloning revealed that the c.834+2T>C mutation induced aberrant transcription and produced a truncated APC protein. ^☆^the stop codon (TAA). mRNA, messenger RNA.

**Table 1:** Summary of variations in all *APC* exons by amplicon sequencing from the proband’s leukocytes and frequency of the mutation (c.834+2T>C) in normal colonic mucosa and a colonic adenomatous polyp using target resequencing

*Site of variant*	*Variant*	*Predicted protein alteration*	*dbSNP141*
*Summary of variations in all APC exons and their boundary regions by amplicon sequencing*			
Intron 7	c. 834+2T>C	Aberrant translation followed by truncation	None
Exon 11	c. 1458T>C	p. Y486Y	rs2229992
Exon 13	c. 1635G>A	p. A545A	rs351771
Intron 14	c. 1958+8T>C	NA	rs62626346
Exon 15	c. 4479G>A	P. T1493T	rs41115
Exon 15	c.5034G>A	p.G1678G	rs42427
Exon 15	c.5268T>G	p. S1756S	rs866006
Exon 15	c.5465T>A	p.V1822D	rs459552
Exon 15	c.5880C>A	p. P1960P	rs465899

*Case*	*Tissue*	*Subject*	*Frequency (%)*	*No. of reads*		
				*Total*	*Ref allele (T)*	*Variant allele (C)*
*Frequency of APC mutation (c.834+2T>C) by target resequencing*						
AGFAP001-1	Blood	1	12.7	206 777	179 342	26 183
	Normal colonic mucosa	1	15.9	3269	2733	521
		2	23.7	18 226	13 756	4328
	Colonic adenomatous polyp	1	37.0	6480	4037	2399
		2	48.0	4553	2329	2184
AGFAP001-2	Blood	1	0.017	294 104	293 795	49
AGFAP001-3	Blood	1	0.013	295 066	294 785	40

Abbreviation: NA, not applicable.
